# Delayed Retroperitoneal Hemorrhage as a Complication of Large-volume Paracentesis

**DOI:** 10.7759/cureus.4167

**Published:** 2019-03-01

**Authors:** Patricia Guzman Rojas, Reetika Sachdeva, Wojtek Blonski

**Affiliations:** 1 Internal Medicine, University of Central Florida College of Medicine, Orlando, USA; 2 Gastroenterology, University of Central Florida College of Medicine, Orlando, USA

**Keywords:** paracentesis, retroperitoneal hemorrhage, cirrhosis

## Abstract

Large-volume paracentesis (LVP) consists of the removal of more than four liters of ascitic fluid. This procedure can cause complications such as hemorrhage, infection, bowel perforation, circulatory failure, or ascitic fluid leakage. The main presentation of paracentesis-induced hemorrhage is abdominal wall hematoma.

An 81-year-old male with a past medical history of obesity and diabetes mellitus presented to our hospital with confusion, new onset black tarry stools, and foul-smelling urine. He was found to be oriented only to person and had abdominal distention with positive fluid wave sign and melanotic stools on rectal exam. Laboratory results elucidated pancytopenia, hypoalbuminemia, elevated aspartate aminotransferase (AST) of 43 U/L, and elevated D-dimer levels. Urinalysis was abnormal, showing >180 white blood cells (WBC) with positive leukocyte esterase and nitrites. Liver ultrasound evidenced cirrhosis. Octreotide drip, ceftriaxone, lactulose, and pantoprazole were initiated for upper gastrointestinal (GI) hemorrhage and cirrhosis. A computed tomography angiogram (CTA) of the chest was positive for bilateral segmental pulmonary embolism, therefore, he also started receiving heparin drip.

On the fifth day of admission, an ultrasound-guided paracentesis was done, with six liters of ascitic fluid removed. On the seventh day of admission, the patient presented acute left flank pain with an associated episode of hypotension and drop in hemoglobin. A CTA of the abdomen showed left retroperitoneal hemorrhage but no signs of active bleeding. Heparin drip was discontinued, and the patient was transferred to the intensive care unit (ICU). The patient’s hemoglobin was stable throughout the days after ICU admission, and he did not require any more transfusions of packed red blood cells. His respiratory status was steady although heparin was discontinued due to a bleeding episode. He was discharged without anticoagulation therapy due to his high risk for rebleeding.

One of the proposed mechanisms leading to variceal bleeding is the rapid decompression of splanchnic circulation due to decreased abdominal pressure. Since the source of bleeding is venous, initially, the patients can be asymptomatic. Treatment can be conservative, surgical or by means of transcatheter interventions.

We would like to emphasize the need for the close monitoring of patients undergoing large-volume paracentesis, especially in the setting of anticoagulation therapy, as survival depends upon early diagnosis and treatment. It is important to mention that international normalized ratio (INR) is neither a reliable anticoagulation test nor a predictive factor of bleeding in cirrhotic patients.

## Introduction

Abdominal paracentesis is a bed-side procedure largely used among cirrhotic patients with refractory ascites. Large-volume paracentesis (LVP) is often performed for symptomatic relief, and this involves the removal of more than four liters of ascitic fluid. This procedure can cause complications such as hemorrhage, infection, bowel perforation, circulatory failure, or ascitic fluid leakage [1-2]. The exact rate of hemorrhage is not known; however, a few studies have reported a rate between 1.7 and 2.9% [3-4]. The main presentation of paracentesis-induced hemorrhage is abdominal wall hematoma; however, pseudoaneurysm and hemoperitoneum are also described in the literature [5].

This case highlights a delayed onset of hemoperitoneum after large-volume paracentesis, which was managed conservatively with the favorable course of the disease.

## Case presentation

An 81-year-old male with a past medical history of obesity and diabetes mellitus presented to our hospital with confusion over the past week, new onset black, tarry stools, and foul-smelling urine over the past day. On examination, the patient was hemodynamically stable and was oriented only to person. However, he had a distended and non-tender abdomen with a positive fluid wave sign. The rectal exam was positive for melanotic stools. Laboratory results elucidated pancytopenia with a hemoglobin of 7.1 g/dL, white blood cells of 3200 K/cm3, platelets of 130,000 k/cm3 and an internal normalized ratio (INR) of 1.22. The chemistry panel showed hypoalbuminemia (2.1 mg/dL) and elevated aspartate aminotransferase (AST) of 43 U/L. Urinalysis was abnormal, showing >180 white blood cell (WBC) with positive leukocyte esterase and nitrites. Liver ultrasound evidenced cirrhosis and reversed portal venous flow without thrombus and ascites. The viral hepatitis panel was negative for hepatitis B or C infection. The patient had a Model for End-Stage Liver Disease (MELD ) score of 9 on admission. Octreotide drip, ceftriaxone, and pantoprazole were initiated for upper gastrointestinal hemorrhage. Due to new-onset decompensated liver cirrhosis associated with encephalopathy, lactulose was started. An elevated D-dimer result (1.06 mg/L) was found, and, for this reason, a computed tomography angiogram (CTA) of the chest was done. This was positive for bilateral segmental pulmonary embolism without features suggesting right heart strain. Therefore, the patient was also started on an unfractionated heparin drip.

On the second day of admission, the patient underwent an esophagogastroduodenoscopy (EGD), showing portal hypertensive gastropathy, with one small area that had a fresh blood clot, which was treated with argon plasma coagulation (APC). There was no evidence of esophageal or gastric varices.

On the fifth day of admission, while anti-factor XA level was 0.53 IU/mL (goal antiXa: 0.3-0.7 IU/mL), a bedside ultrasound-guided paracentesis was done. Six liters of ascitic fluid were removed for diagnostic and therapeutic purposes and this was compatible with portal hypertension, with a serum-ascites albumin gradient (SAAG) of 1.8 g/dL and total protein of 2.0 g/dL. Cell count showed spontaneous bacterial peritonitis (with 365 polymorphonuclear cells/mm^3^), which was treated with intravenous antibiotics. Ascitic fluid cultures showed Escherichia coli, sensitive to ceftriaxone.

**Figure 1:**
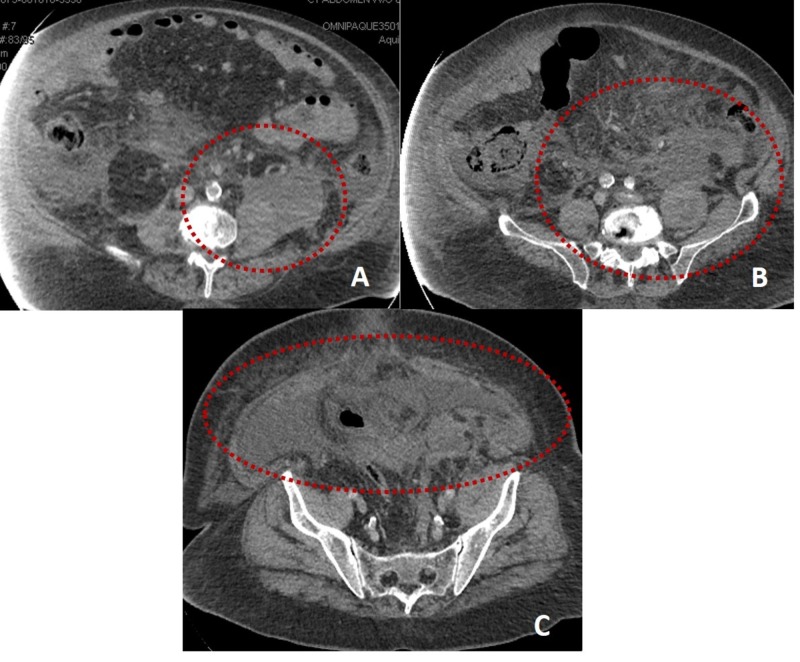
Computed tomography of the abdomen showing hyperdense fluid primarily within the left retroperitoneum, with extension into the lower right retroperitoneum. Hemorrhage centered in the left posterior pararenal compartment with possible extension into the substance of left psoas muscle. Panel A-C: Axial view showing different levels of the hemorrhage

**Figure 2:**
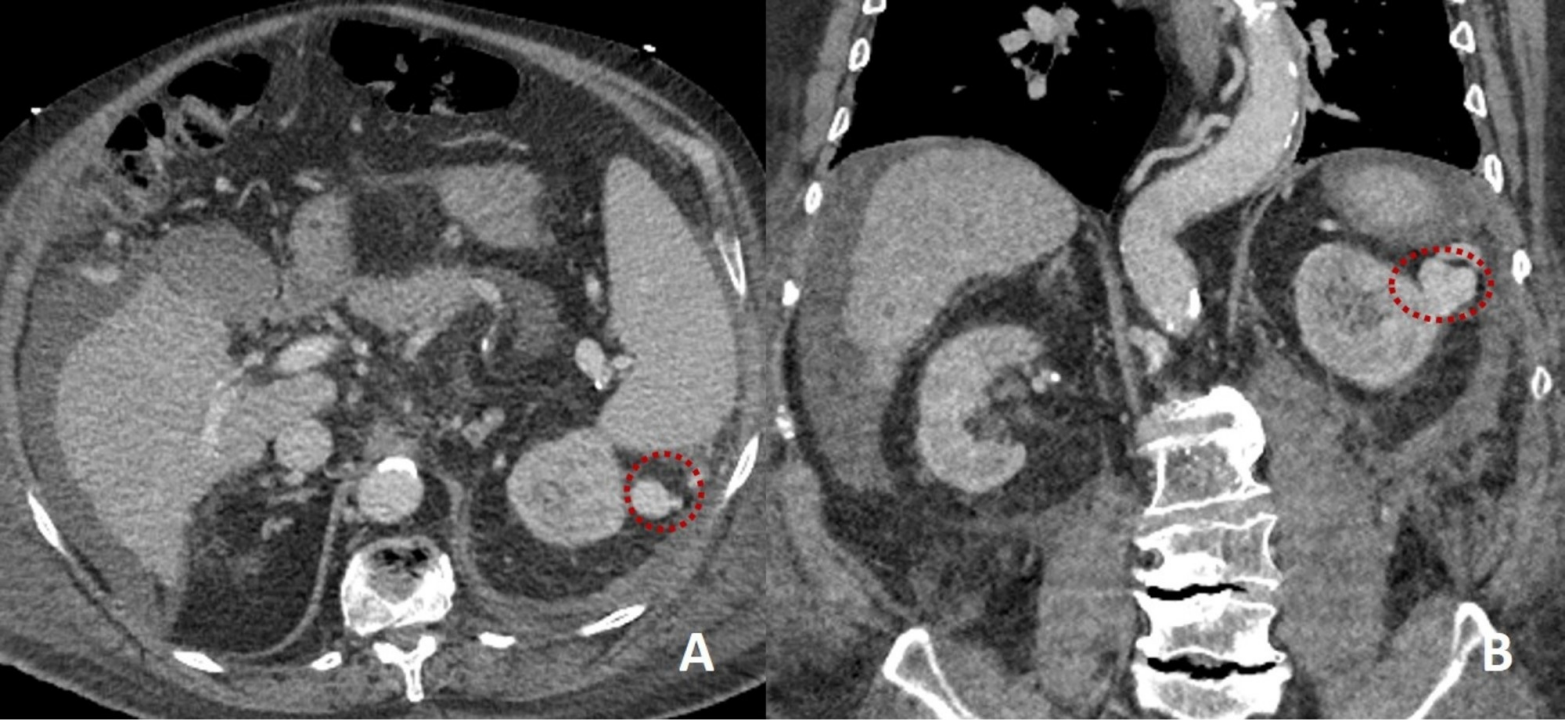
Computed tomography of the abdomen showing a very tortuous splenorenal shunt, which coursed about the spleen laterally and possessed a notable dilated varix maximally measuring 22 mm. Panel A showing axial view and Panel B showing coronal view

On the seventh day of admission, the patient presented acute left flank pain with an associated episode of hypotension. Hemoglobin levels dropped to 5.4 g/dL from 8.2 g/dL the day before. CTA abdomen showed a left retroperitoneal hemorrhage extravasating to extraperitoneal areas (Figure 1). There was a suspicion for portosystemic varices within the peritoneum and mesentery. Another finding was a splenorenal shunt measuring 2.2 centimeters (Figure 2). There were no signs of active hemorrhage. Anti-XA level was 0.51 IU/mL. Heparin drip was discontinued, and the patient was transferred to the intensive care unit (ICU) where he was treated conservatively with fluid resuscitation and blood transfusions as needed for a goal of hemoglobin more than 7 g/dL.

The patient’s hemoglobin was stable throughout the days after ICU admission, and he did not require any more transfusions of packed red blood cells. His respiratory status was steady despite the discontinuation of anticoagulation with heparin. He was discharged without anticoagulation therapy due to a high risk for rebleeding.

## Discussion

Hemorrhage following a paracentesis can be acute or delayed. The patient had a delayed retroperitoneal hemorrhage, with onset two days after the completion of paracentesis that demonstrated clear ascitic fluid.

One of the proposed mechanisms leading to variceal bleeding is the rapid decompression of splanchnic circulation due to decreased abdominal pressure [1]. This can lead to an increase in portal pressure and blood flow through the portal-systemic shunts, causing varices to rupture and bleed. The patient's retroperitoneal hemorrhage was thought to be secondary to the splenorenal shunt, versus other retroperitoneal portosystemic collateral pathways, while he was on therapeutic anticoagulation therapy.

Since the source of bleeding is venous, initially, the patients can be asymptomatic. However, when the volume of blood is large enough to cause symptoms, vague abdominal pain is commonly described [2]. However, peritoneal signs are rare. Given this subtle presentation, possible risk factors should be identified in patients undergoing LVP.

Lin et al. found that the fibrinogen level in patients with a MELD score higher than 25 was a predictor factor for hemorrhagic complications after paracentesis [4]. Furthermore, Pache et al. reported that an elevated international normalized ratio (INR) and/or thrombocytopenia are factors that do not correlate with hemorrhagic complications, although patients with higher MELD and Child-Pugh scores had higher rates of hemorrhage [6]. This statement is also confirmed by Tripodi et al. who concluded that prothrombin time (PT) and INR are unreliable tools to evaluate the risk of bleeding in patients with cirrhosis [7]. It is also important to emphasize that there is no strong evidence that supports blood products transfusion in patients with coagulopathy, undergoing a paracentesis. Furthermore, according to the American Association of Study of Liver Diseases (AASLD) guidelines, there is no specific cutoff of coagulation parameters to avoid paracentesis [8]. The patient was receiving a heparin drip due to underlying pulmonary embolism, which could have predisposed him to hemorrhage.

Hemoglobin levels should be closely monitored several days after a procedure in patients with the previously mentioned risk factors. If a noticeable drop is detected, diagnostic paracentesis should be recommended. If the ascitic fluid is positive for blood, abdominal imaging with CT with contrast or CTA must be performed to confirm hemorrhage [6,9].

There are different approaches to the management of this entity: Conservative treatment, surgery (laparotomy), and/or transcatheter interventions like transjugular intrahepatic portosystemic shunting (TIPS), embolization, or coiling. In the presented case, interventional radiology was closely involved and coiling of the splenorenal shunt was a reviewed possibility if the bleeding continued. Mortality associated with hemorrhage has been found to be as high as 75% in patients undergoing surgical treatment, however, this is decreased when the approach is done by embolization (33%) [5].

## Conclusions

We would like to emphasize the need for the close monitoring of patients undergoing large-volume paracentesis, especially in the setting of anticoagulation therapy, as survival depends upon early diagnosis and treatment. It is important to mention that international normalized ratio (INR) is neither a reliable anticoagulation test nor a predictive factor of bleeding in cirrhotic patients.
